# Shotgun proteomic analysis of nanoparticle-synthesizing *Desulfovibrio alaskensis* in response to platinum and palladium

**DOI:** 10.1099/mic.0.000840

**Published:** 2019-07-30

**Authors:** Michael J. Capeness, Lisa Imrie, Lukas F. Mühlbauer, Thierry Le Bihan, Louise E. Horsfall

**Affiliations:** ^1^​ Institute of Quantitative Biology, Biochemistry and Biotechnology/CSEC, School of Biological Sciences, University of Edinburgh, Edinburgh, EH9 3FF, UK; ^2^​ EdinOmics, SynthSys, CH Waddington Building, Max Born Crescent, The King's Buildings, Edinburgh, EH9 3BF, UK​; ^3^​ Currently: Rapid Novor, Inc., Kitchener, Ontario N2G 4P3, Canada

**Keywords:** nanoparticle, platinum, palladium, *Desulfovibrio*, proteomics

## Abstract

Platinum and palladium are much sought-after metals of critical global importance in terms of abundance and availability. At the nano-scale these metals are of even higher value due to their catalytic abilities for industrial applications. *Desulfovibrio alaskensis* is able to capture ionic forms of both of these metals, reduce them and synthesize elemental nanoparticles. Despite this ability, very little is known about the biological pathways involved in the formation of these nanoparticles. Proteomic analysis of *D. alaskensis* in response to platinum and palladium has highlighted those proteins involved in both the reductive pathways and the wider stress-response system. A core set of 13 proteins was found in both treatments and consisted of proteins involved in metal transport and reduction. There were also seven proteins that were specific to either platinum or palladium. Overexpression of one of these platinum-specific genes, a NiFe hydrogenase small subunit (Dde_2137), resulted in the formation of larger nanoparticles. This study improves our understanding of the pathways involved in the metal resistance mechanism of *Desulfovibrio* and is informative regarding how we can tailor the bacterium for nanoparticle production, enhancing its application as a bioremediation tool and as a way to capture contaminant metals from the environment.

## Introduction

There is an increasing demand for expensive platinum-group metals. Amongst other applications, they are used for automotive catalytic converters from which platinum and palladium escape via the exhaust, making them a source of environmental pollution [1]. Although heavy metal ions are toxic to most bacteria, highly resistant strains are available that absorb them and reduce them to the metallic state. This results in the formation of nanoparticles that can be harvested to provide a source of these valuable metals. Furthermore, nanoparticles from bacteria have important biotechnological applications, for example, as chemical catalysts. Not only does biological recovery from environmental sources offer an attractive way to recover them from dilute solutions, but it also provides a mechanism for decontaminating polluted environments. Prokaryotes have been used to remediate metal ions from solution for decades: mostly high value metals such as gold, platinum and silver have been targeted [2, 3].

The potential uses of nanoparticles are expanding, with platinum or arsenic nanoparticles being used in cancer treatments [4, 5], silver nanoparticles being used as antimicrobials [6], and palladium nanoparticles being used as catalysts in fuel cells [7] and to treat water contaminated with pharmaceuticals [8]. Tailoring these nanoparticles for their implementation in industrial or medical applications is vital, but so far there has been little standardization with regard to the size of nanoparticles formed [9]. As different sized nanoparticles have different catalytic properties, their use has been limited. Bacteria potentially represent a genetically programmable method for the customization of nanoparticle size and morphology for specific purposes. Bioremediation by bacteria also involves mild conditions and ambient temperatures, thus avoiding the disadvantages of chemical recovery that requires the use of high temperatures or hazardous chemicals.

Anaerobic, sulphate-reducing bacteria of the genus *Desulfovibrio* are amongst the most widely used bacteria to remediate metal ions. They reduce multi-valent metal ions to zero-valent or bi-elemental nanoparticles. The list of known metals that it can process is ever increasing and currently includes chromium, magnesium, iron, technetium, uranium and nickel [10–15], and also platinum and palladium, of which palladium is the most studied. The *Desulfovibrio* sp. are ideally suited for further analysis as they are already highly tolerant to platinum and palladium ions, and can survive up to 2 and 5 mM, respectively [15]. They reduce these metals at incredible speed, with up to 100 % of Pd^2+^ being converted to Pd(0) within 5 min [7], and also are able to synthesize bi-metallic nanoparticles containing both elemental palladium and platinum [16].

Only fragmentary information is available at the genetic level concerning the *Desulfovibrio* genes required for heavy metal ion reduction. One exception is *cycA* from *Desulfovibrio vulgaris* Hildenborough, which encodes a tetraheme cytochrome *c*_3_ that is involved in uranium reduction *in vitro* [17]. The NiFe hydrogenases of *Desulfovibrio fructosovorans* have been shown to be involved in technetium reduction [18]. They similarly have a role, along with an Fe-containing hydrogenase, in Pd(0) deposition and clustering in both the periplasm [19] and on cellular membranes [20], while more recently they have been implicated in having a role in palladium deposition in *Shewanella* [21]. While important for the anaerobic reduction of palladium by *Desulfovibrio* sp., they are not required for the aerobic reduction of palladium by *Escherichia coli* [22]. Large gaps remain in our knowledge of the proteins involved in metal ion reduction. In many previous studies washed bacteria were incubated in the presence of the target metal in buffer rather than in substrates required for growth, making downstream analysis and isolation easier. We therefore designed experiments to identify changes in the proteome of *Desulfovibrio alaskensis* strain G20 during incubation with Pt^4+^ or Pd^2+^ solutions under conditions used in previous studies both by ourselves and many other research teams. One of the genes implicated in Pt^4+^ reduction was cloned into an expression vector in a proof-of-principle experiment designed to demonstrate that overproduction of the gene product results in changes in the physical properties of the nanoparticles formed.

There are few previous studies of proteomics in *Desulfovibrio* sp. to elucidate the effect of metals on the organism and to discern the pathways of metal resistance; most proteomic studies have only targeted single genes for mutation [18, 23]. Proteomics has, however, been used to find new pathways induced by metal toxicity in a wide variety of other metal-tolerant bacteria, including cadmium, zinc and copper tolerance in *Pseudomonas fluorescens* [24], radium-induced responses in *Serratia marcescens* [25] and arsenic tolerance in *Klebsiella pneumonia* [26]. Proteomics is thus a great tool for finding unknown proteins in the pathways of understudied organisms that may show adaption to metal tolerance. Furthermore proteomics, unlike transcriptomics, shows the proteins that are more directly involved in response to the treatment and are not subject to transcription regulation, and so it avoids the tendency of the the latter technique to result in a large discrepancy between the signals detected and the effects.

## Methods

### Growth of bacterial strains

*D. alaskensis* G20 was purchased from Deutsche Sammlung von Mikroorganismen und Zellkulturen (DSMZ) and grown in Postgate medium C (PGMC) using lactate as a carbon source [27]. Cultures were grown and manipulated in an anaerobic chamber (Don Whitley) at 30 °C in an atmosphere of 10 % CO_2_, 10 % H_2_ in N_2_. For strains carrying the pMO9075 plasmid the medium was supplemented with 100 µg ml^−1^ spectinomycin (Cambridge Bioscience).

### Platinum and palladium treatment

Bacteria were grown in Postgate medium C to an OD_600_ of 1.0 and centrifuged for 10 min at 4000 r.p.m. The cell pellets were washed three times in 10 mM MOPS (pH 7.0) and resuspended to an OD_600_ of 1.0 in 10 mM MOPS. Solutions of PtCl_4_ or Na_2_PdCl_4_ were then added to a final concentration of 2 mM. The cells in the presence of palladium and platinum and an unsupplemented control suspension were incubated anaerobically at 30 °C. After 2 h, samples were centrifuged as before and the supernatant was removed. The pellets were either snap-frozen and stored at −80 °C or used immediately for transmission electron microscopy (TEM) analysis. Controls of the buffer alone with either platinum or palladium were also carried out for subsequent microscopy analysis, although the formation of nanoparticles was not observed.

### Proteomic analysis: trypsin digestion, and data analysis

Cells were disrupted in 8 M urea and the total protein was assayed using a Bradford kit (Biorad, UK). One milligram of protein per sample was digested with trypsin as described previously [28]. Briefly, samples were diluted to a final concentration of 2 M urea with 25 mM ammonium bicarbonate and 5 mM DTT. After incubation for 30 min at room temperature, iodoacetamide was added to a final concentration of 12.5 mM. Trypsin (10 µg; Worthington) was added and the sample was digested overnight at room temperature. The samples were cleaned with 25 mg of Bond Elut LMS (Agilent Technologies). Peptides eluted with acetonitrile were aliquoted, dried under low pressure and stored at −20 °C until analysis. Prior to LC-MS (liquid chromatography–mass spectrometry) analysis, 4 µg of the samples were reconstituted in 12 µl of loading buffer (0.05 % trifluoroacetic acid in water) and analysed by capillary-HPLC-MS/MS (high performance liquid chromatography-tandem mass spectrometry) on an on-line system consisting of a 1200 binary HPLC micro-pump system (Agilent Technologies) coupled to a hybrid LTQ-Orbitrap XL instrument (Thermo Fisher) on a 140 min gradient.

**Fig. 1.**
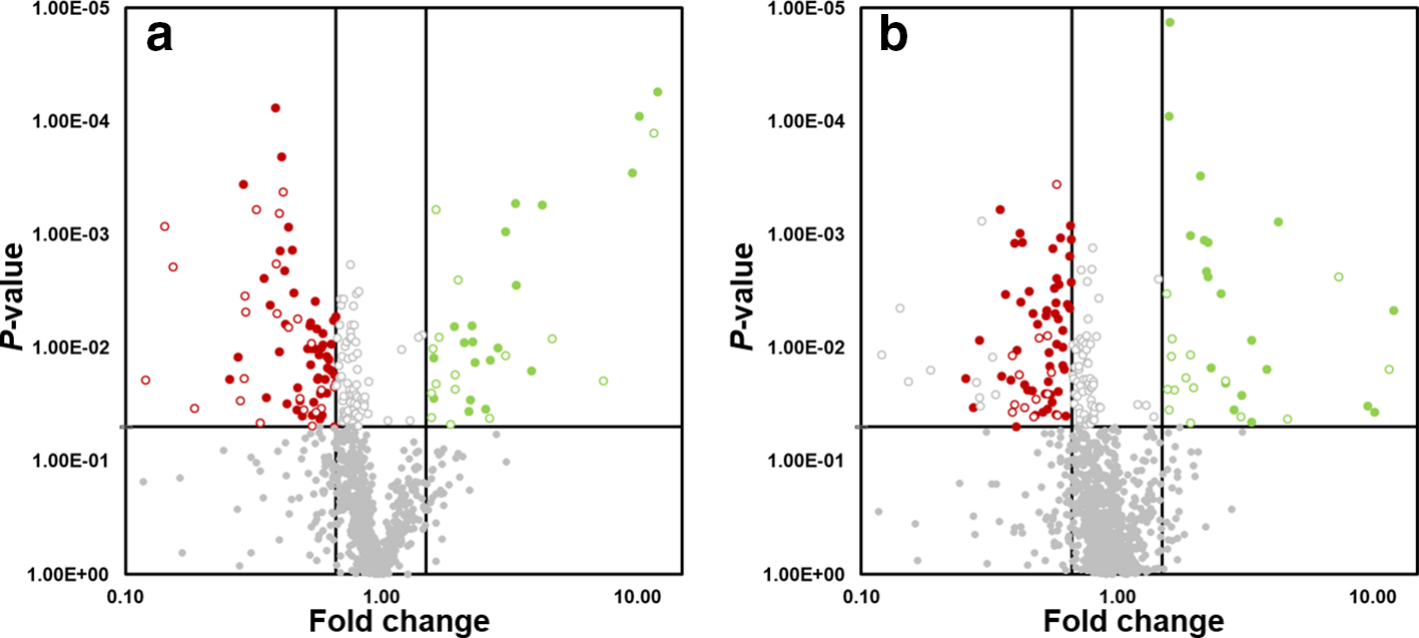
Volcano plot for the data generated in the shotgun proteomics for the platinum (a) and palladium (b) datasets. The full red circles indicate the proteins that are less abundant and the full green circles represent those that are more abundant (both of which have a probability >0.05). The grey circles indicate proteins that are not significantly different across the three different datasets. The empty circles represent the proteins that are significant but are only represented by a single peptide.

Tandem mass spectrometry (MS/MS) data were analysed using MASCOT version 2.4 (Matrix Science Ltd, UK) against the 3258 sequences in the *D. alaskensi *s G20 genome from http://genome.ornl.gov/microbial/ddes [29]. The Mascot search parameters were set at two missed cleavage sites. Allowances were made for variable methionine oxidation and fixed cysteine carbamidomethylation in all searches. The precursor mass tolerance was fixed at 7 p.p.m and the MS/MS tolerance was set to 0.4 amu. The significance threshold was set at 0.05 and an additional cutoff peptide score of 20. Progenesis (Nonlinear Dynamics, UK) was used for label-free quantitation. Only peptides that were not shared between different proteins were used for quantification. Data for a sub-set of MS/MS peaks with positive charges of 2, 3, or 4 were extracted from each LC-MS run and the median global ion intensities were extracted for normalization. The abundance of each protein was calculated from the sum of the intensities of unique peptides with positive charges of 2, 3, or 4. Because the method of detection can generate a significant number of near-zero measurements for which a standard log transformation is not ideal, the calculated protein abundances were transformed using an ArcSinH function. The within-group means were calculated to evaluate the fold change and the ArcSinH-transformed data were then used to calculate the *P*-values using one-way analysis of variance (ANOVA). Proteins were only considered to be differentially expressed if at least two peptides were detected with an absolute ratio of at least 1.5-fold greater abundance or 0.667 less abundance and a probability of false discovery of *P*<0.05, as illustrated in Fig. 1. The mass spectrometry proteomics data have been deposited to the ProteomeXchange Consortium via the PRIDE partner repository with the dataset identifiers PXD004457 and 10.6019/PXD004457 [30]. The data are also available in the Supplementary Material Dataset (available in the online version of this article). The functions of the proteins highlighted in this study were predicted using a combination of literature searches and cross-referencing with the KEGG (http://www.kegg.jp/kegg/kegg2.html), STRING (http://string-db.org/), Pfam (http://pfam.xfam.org/) and UniProt databases (http://www.uniprot.org/). The presence of platinum group metals in RNA preparations prevented us from using quantitative real-time PCR to confirm changes in the expression of genes for the proteins highlighted at the transcription level.

### Construction of pMO-2137

The gene for the Dde_2137 subunit of the nickel–iron hydrogenase was cloned into the expression vector pMO9075 using a standard Phusion PCR protocol [31]. The forward and reverse primers were dde_2137F 5′-CAGTACTCTGCAGATCTGATCCTTTCATTGC-3′ and dde_2137R 5′-TGCATGCCAATTCGTTTCGGTACCGGC-3′. The restrictions sites for *Sca*I and *Sph*I are underlined. The PCR product was digested with *Sca*I and *Sph*I and ligated into pMO9075 using these restriction sites. The sequence was inserted downstream of the promoter of the kanamycin resistance gene in pMO9075, the expression from which has been shown to complement mutations in *Desulfovibrio* sp.

The pMO-2137 and empty pMO9075 plasmids were transformed into *D. alaskensis* made competent using an adaptation of Li and Krumholz’s method [32]. Briefly, cultures at an OD_600nm_ of 0.6–0.8 were centrifuged, washed twice in 400 mM sucrose and 1 mM MgCl_2_, and finally resuspended in 1/10 of the original volume of sucrose solution. One microgram of the plasmid was then added. After 30 min on ice, the suspension was electroporated at 1.5 kV. After electroporation, 1 ml of PGMC broth was added immediately to allow the cells to recover at 30 °C. After 4 h, cells were plated onto PGMC agar containing 100 µg ml^−1^ of spectinomycin. After 7 days at 30 °C, transformants were purified and screened by plasmid isolation for the presence of the construct. For comparative analysis of nanoparticles produced by bacteria transformed with either the empty plasmid control or the plasmid carrying the extra copy of the dde_2137 gene, the OD_600nm_ of the cells was matched before the addition of platinum ions.

### TEM

Samples were drop-cast onto a 200 mesh copper grid with a formvar coating (TAAB). The sample was allowed to settle for 5 min before excess liquid was removed using filter paper. The grid was then loaded into a JEOL JEM-1400 Plus electron microscope and imaged using a GATAN OneView Camera. Dm3 were processed using ImageJ software (http://imagej.nih.gov/ij/) and nanoparticles were sized using the measure tool across the longest width of each nanoparticle. A total of 48 images were used (18 for the control, 30 for pMO-2137) arising across independent TEM sessions and biological replicates.

### Energy-dispersive X-ray spectroscopy

Samples prepared as above for conventional TEM were drop cast onto holey carbon nickel grids (Agar Scientific). The samples were analysed using a JOEL JEM 2011 TEM fitted with an ISIS system and viewed at an accelerating voltage of 200 kV.

## Results and discussion

### Overview of the proteomic data

Transfer of a bacterial culture from a growth medium into buffer containing toxic levels of a heavy metal results in an almost total cessation of growth. Amongst many other consequences, this results in ribosome degradation and therefore decreased demand for stable RNA and ribosomal proteins (Table 1). The degradation products can then be recycled for the synthesis of components that are beneficial for the stress response. Conversely, incubation in a buffer in the absence of growth nutrients inevitably limits the ability of bacteria to respond to stress. Any increased protein synthesis is therefore likely to be limited to the synthesis of those proteins that increase survival of the stress imposed. These were expected to be proteins involved in the reduction of the specific metal ions, in transporting them out of the cell, or in a more general response to stress. A total of 1061 proteins from the *D. alaskensis* G20 potential proteome were identified by shotgun label-free proteomics, representing 32 % of the global proteome (see the Supplementary Material Dataset for a complete list). In terms of coverage, this is in keeping with other proteomic studies concerning metal trials in bacteria, including those for *Desulfovibrio* sp. [23, 33].

**Table 1.** Proteins significantly more abundant (>1.5 fold) in the presence of platinum and palladium and their overlap between datasets

Platinum and palladium			Platinum only			Palladium only		
**Protein ID**	**Predicted function**	**Average fold change**	**Protein ID**	**Predicted function**	**Average fold change**	**Protein ID**	**Predicted function**	**Average fold change**
**Metal sequestering/transport**			**Cytochromes/hydrogenases**			**Other**		
Dde_0155	Molybdenum ABC transporter	3.17	Dde_2642	4Fe–4S ferredoxin iron–sulfur binding domain-containing protein	2.55	Dde_0444	Peptidase M16 domain protein	2.17
Dde_0186	ABC-type transporter, periplasmic subunit family 3	2.85	Dde_2137	Periplasmic (NiFe) hydrogenase, small subunit, isozyme 1	2.66	Dde_2315	Hypothetical protein Dde_2315	2.12
Dde_0258	Extracellular ligand-binding receptor	2.19	Dde_2138	Cytochrome-*c*_3_ hydrogenase	1.60	Dde_3028	Carbon-monoxide dehydrogenase, catalytic subunit	1.68
**Export mechanism**			**Other**			Dde_1618	NAD(P)H dehydrogenase (quinone)	1.67
Dde_3627	Outer-membrane efflux protein	5.62	Dde_0168	ABC-type transporter, periplasmic subunit family 3	3.86	Dde_2641	Hybrid cluster protein	1.64
Dde_1415	Type I secretion outer-membrane protein, TolC family	2.78	Dde_1279	Methyl-accepting chemotaxis sensory transducer with Pas/Pac sensor	2.23	Dde_2200	1-deoxy-d-xylulose-5-phosphate synthase	1.63
**Cytochromes/hydrogenases**			Dde_1545	Single-strand binding protein	2.11	Dde_1699	Processing peptidase	1.58
Dde_2934	4Fe–4S ferredoxin iron–sulfur binding domain-containing protein	4.00	Dde_1214	Rubrerythrin	1.94			
Dde_2933	Molybdopterin oxidoreductase	3.48						
**Other**								
Dde_0806	Rhodanese-like protein	11.71						
Dde_1442	Signal peptide peptidase SppA, 36K type	11.02						
Dde_3216	Phosphatidylserine decarboxylase-related protein	9.53						
Dde_0194	Stress-responsive alpha-beta barrel domain-containing protein	4.87						
Dde_1010	Hypothetical protein Dde_1010	3.06						
Dde_2342	Glyceraldehyde-3-phosphate dehydrogenase, type I	2.11						

Using a significance threshold of 1.5-fold change and a *P*-value below 0.05, the abundance of 46 proteins decreased during incubation in the presence of palladium and platinum ions, of which 24 were in the platinum dataset and 22 were in the palladium dataset, with 20 proteins found in both datasets. Only 20 proteins were found to be more abundant in cells treated with palladium compared with untreated cells. Similarly, 20 proteins were also more abundant in cells treated with platinum, 13 of which were also induced during incubation with palladium ions. Consequently, seven of the more abundant proteins were unique to each dataset (Fig. 1).

### Proteins less abundant in both the platinum and palladium datasets

As expected, the proteins found in lower amounts in both datasets are predominantly those involved in RNA transcription and translation. These include both large and small ribosomal subunit proteins (L32, L29, S187, S12) as well as transcription regulators of the TraR/DskA and MucR families (Table 2). The S12 ribsomal protein in particular has previously been shown to be less abundant in proteomic studies in response to platinum (cisplatin) in *E. coli* [34]. One hypothetical protein identified (Dde_0492) is predicted to be involved in valyl-tRNA synthesis while another (Dde_1811), of unknown function, are only conserved among the *Desulfovibrio* sp. and SRB deltaproteobacteria respectively. The least abundant protein relative to the control was the periplasmic Fe hydrogenase small subunit (Dde_2280); this and Dde_2274 are potential hydrogenases that appear not to be involved in the reduction of either platinum or palladium. This suggests that there might be a separate hydrogenase and other electron transfer proteins that are specific for platinum and palladium reduction. Also in the less-abundant dataset are proteins involved in energy production and metabolism, such as oxidoreductases (Dde_0584, Dde_1113, Dde_3240, Dde_2272 and Dde_1638), one ATPase subunit (Dde_0990) and the prokaryotic molybdopterin-containing oxidoreductase (Dde_2274).

**Table 2.** Proteins significantly less abundant (<0.667-fold) in the presence of either platinum and palladium and their overlap between datasets

Platinum and palladium			Platinum only			Palladium only		
**Protein ID**	**Annotation**	**Average Fold Change**	**Protein ID**	**Annotation**	**Fold change**	**Protein ID**	**Annotation**	**Fold Change**
**Energy production/metabolism**			**Energy production/metabolism**			**Energy production/metabolism**		
Dde_2280	Periplasmic Fe hydrogenase small subunit	0.29	Dde_2018	HesB/YadR/YfhF-family protein	0.41	Dde_2273	Periplasmic (Sec) triheme cytochrome c	0.53
Dde_3667	Flavodoxin	0.35	Dde_0445	ABC transporter related protein	0.42	Dde_1114	Hypothetical protein Dde_1114	0.63
Dde_0584	NADH : quinone oxidoreductase subunit RnfE	0.45	Dde_2669	Ferrous iron transport protein B	0.53	Dde_2271	Inner-membrane protein binds 2 heme b	0.63
Dde_1113	Quinone-interacting membrane-bound oxidoreductase	0.5	Dde_2670	Ferrous iron transporter component feoA	0.54	Dde_1111	Quinone-interacting membrane-bound oxidoreductase	0.64
Dde_3240	Protein of unknown function DUF224	0.55	Dde_1633	Gamma-glutamyl phosphate reductase	0.55	Dde_3604	d-lactate dehydrogenase (cytochrome)	0.65
Dde_2272	Hdr menaquinol oxidoreductase	0.56	Dde_2979	Carbonic anhydrase	0.57	Dde_2201	Polyprenyl synthetase	0.65
Dde_0990	H^+^ transporting two-sector ATPase B/B' subunit	0.56	Dde_3520	Molybdate-transporting ATPase	0.58	Dde_3708	Transmembrane complex, integral membrane protein	0.65
Dde_1638	2-oxoglutarate synthase	0.58	Dde_2341	Fructose-1,6-bisphosphate aldolase, class II	0.66	Dde_1112	Quinone-interacting membrane-bound oxidoreductase	0.65
Dde_2274	Molybdopterin-containing oxidoreductase family, iron–sulfur-binding subunit	0.59	**Transcription/translation**			**Transcription/translation**		
**Transcription/translation**			Dde_1173	Protein of unknown function DUF306 Meta and HslJ	0.54	Dde_2637	Ribosomal protein L35	0.51
Dde_4007	50S ribosomal protein L32	0.31	Dde_2982	Ribosomal protein L31	0.66	Dde_1095	Ribosomal protein L19	0.52
Dde_0492	Hypothetical protein Dde_0492	0.45	**Cell integrity/maintenance**			Dde_0327	Response regulator receiver protein	0.55
Dde_2249	Ribosomal protein L29	0.47	Dde_1413	Organic solvent tolerance protein	0.01	Dde_0346	Transcriptional regulator, MucR family	0.55
Dde_2345	Transcriptional regulator, TraR/DksA family	0.47	Dde_0453	MotA/TolQ/ExbB proton channel	0.28	Dde_0793	RNP-1-like RNA-binding protein	0.56
Dde_1411	Ribosomal protein S18	0.53	Dde_1368	Outer-membrane protein assembly complex, YaeT protein	0.42	Dde_2254	Ribosomal protein L2	0.57
Dde_2238	Ribosomal protein L15	0.56	Dde_2153	MotA/TolQ/ExbB proton channel	0.43	Dde_2233	30S ribosomal protein S11	0.57
Dde_2613	Transcriptional regulator, MucR family	0.57	Dde_1587	Hypothetical protein Dde_1587	0.52	Dde_2127	Ribosomal 5S rRNA E-loop-binding protein Ctc/L25/TL5	0.58
Dde_2263	Ribosomal protein S12	0.6	Dde_3632	Tol-Pal system beta propeller repeat protein TolB	0.58	Dde_2608	Ribosomal protein S9	0.58
**Cell integrity/maintenance**			Dde_3641	Sulphate transporter	0.61	Dde_2607	Ribosomal protein L13	0.59
Dde_2145	Tol-pal system protein YbgF	0.46	Dde_1689	OmpA/MotB domain protein	0.61	Dde_2255	Ribosomal protein L25/L23	0.61
**Other**			Dde_2066	Thioredoxin reductase	0.63	Dde_2253	Ribosomal protein S19	0.63
Dde_0030	Hypothetical protein Dde_0030	0.44	**Other**			Dde_2232	Ribosomal protein S4	0.64
Dde_1811	Hypothetical protein Dde_1811	0.48	Dde_0448	ABC-type transporter, periplasmic subunit	0.24	Dde_1133	Ribosomal protein S2	0.64
			Dde_2440	Putative lipoprotein	0.37	Dde_3162	Translation initiation factor IF-2	0.66
			Dde_0493	Hypothetical protein Dde_0493	0.4	Dde_1175	RNP-1 like RNA-binding protein	0.66
			Dde_2152	Hypothetical protein Dde_2152	0.43	Dde_3166	Ribosomal protein S15	0.66
			Dde_0316	Hypothetical protein Dde_0316	0.51	Dde_1025	GrpE protein	0.66
						Dde_0507	Ribosomal protein S1	0.67
						**Cell integrity/maintenance**		
						Dde_2278	Biotin and thiamine synthesis-associated	0.4
						Dde_2294	(Acyl-carrier-protein) S-malonyltransferase	0.59
						**Other**		
						Dde_1571	Response regulator receiver modulated CheB methylesterase	0.54
						Dde_1694	Response regulator receiver protein	0.58

### Platinum-specific proteins

Proteins involved in cell integrity and maintenance as well as those required for growth dominated the list of those that were less abundant following incubation with Pt^4+^ ions. Proteins such as Dde_1413, Dde_0453, Dde_1368, Dde_2153, Dde_3632, Dde_1689 and Dde_1131 are predicted to be involved in membrane biogenesis and integrity, which is consistent with decreased synthesis of new membranes due to cessation of growth. This list also includes other proteins with a common role: Dde_3488, Dde_3490, Dde_3567, Dde_2763, Dde_3070, Dde_3703 and Dde_2744 are all predicted to be involved in amino acid synthesis. Again, this supports the suggestion that, due to the limited media in which the NPs are produced [MOPS, (3-(N-morpholino)propanesulfonic acid) buffer], there is limited synthesis of new proteins. Other less abundant proteins, Dde_2018, Dde_0445, Dde_2669 and Dde_2670, are required for iron or haem uptake, possibly as a risk reduction strategy due to their general metal binding and transportation ability.

### Palladium-specific proteins

The palladium dataset is dominated by ribosomal subunit proteins both small (Dde_2233, Dde_2608, Dde_2253, Dde_2232 and Dde_1133) and large (Dde_2637, Dde_1095, Dde_2254, Dde_2607 and Dde_2255). There are also other proteins involved in transcription and translation, such as the rRNA E-loop-binding protein (Dde_2127), the response regulator and CheY-like protein (Dde_0327), the translation initiation factor (Dde_3162) and the MucR transcriptional regulator (Dde_0346). Decreased levels of these proteins would have a global effect on transcription and translation and silencing their expression would arrest the cell cycle. The next largest group that are less abundant specifically in Pd^2+^-treated bacteria are proteins required to provide metabolites for energy production and growth. The proteins Dde_1111 to 1114 (with Dde_1113 found to be downregulated following both platinum- and palladium-specific treatment) are products of a group of contiguous genes encoding part of the membrane-bound Qmo electron transport complex. The operon is vital to the growth of *D. vulgaris* Hildenborough on lactate–sulphate media [31, 35]. Downregulation of this operon would make sense, as the cells have gone from a lactate–sulphate-containing growth medium to a very minimal one.

In conclusion, for the less-abundant dataset, while many of the candidate proteins are unsurprising and were expected to be lower in abundance (those involved in transcription, transcription and energy metabolism), a separate group of proteins does seem to be identified, depending upon the metal used in the treatment, much like with those proteins that are more abundant. The metal treatment also appears to have a significant effect on the cells in comparison to the control dataset (buffer only). Different proteins are highlighted in each case, and although many of these proteins are expected, as a result of the cells being shifted from growth media (PGMC or MOPS), the added presence of the metal in the treatments has reduced the presence of those proteins to a level that is far below that of the control experiment, such that they do appear to be of significance in this proteomic study.

### Proteins that are more abundant following incubation with Pt^4+^ or Pd^2+^ ions

Three of the proteins showing increased abundance in both datasets included a ferredoxin and a molybdoprotein, MopB, encoded by adjacent genes on the chromosome, and the rhodanese Dde_0806. Evidence that these proteins are required for metal ion reduction includes a report that a *mopB* mutant is unable to grow on hydrogen or formate as the electron donor [36], and that a rhodanese mutant is also unable to use hydrogen as an electron donor [37]; both of these mutations lead to a sensitivity to metal ions, as the mutants can no longer reduce the metals as effectively. More recently, it was also shown that molybdoproteins are required for Pd^4+^ reduction by *E. coli* [22].

**Fig. 2.**
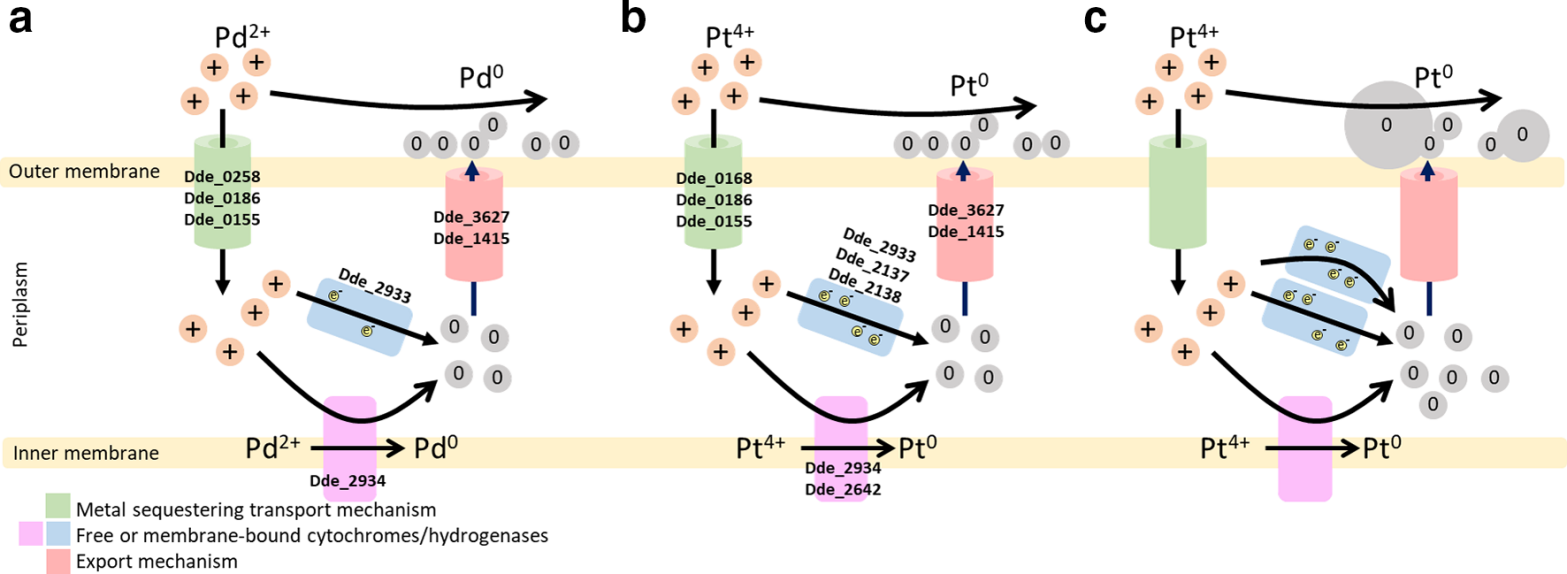
Proposed mechanism of Pd and Pt nanoparticle synthesis in *D. alaskensis* G20 based on the proteomic data presented here for palladium (a) and platinum (b). Note the overlapping of proteins found in each of the individual metal datasets found in the ESI (table 1). (c) Predicted production of increased size platinum nanoparticles due to increased levels of Dde_2137. Adapted and expanded from [15].

Five other proteins encode putative ABC-type transportation proteins, Dde_0155, Dde_0186 and Dde_0258, an efflux pump, Dde_3627, and Dde_1010, which potentially encodes an outer-membrane porin. Also in this group is a member of the TolC family of type I secretion systems (Dde_1415) (table 1). It is interesting that efflux (but not influx) machinery is conserved between the metal treatments. Based on this, a model for the import, reduction and efflux of both the Pd^2+^ and Pt^4+^ ions with the proteins highlighted in this study (both specific and shared by each metal dataset) is presented in Fig. 2.

The remaining five proteins common to both datasets all play more general roles in the response to stress. They include Dde_0194, which is an alpha-beta barrel protein, and Dde_1442, an ATP-dependent peptidase likely to be involved in the degradation of proteins that have misfolded due to the presence of the heavy metals [38]. Also in this group is Dde_3216, a protein predicted to be involved in lipid metabolism and repair: such proteins would have a role in managing the effects heavy metals or nanoparticles have on lipid membranes. Lipid peroxidation in particular is one of the means by which TiO_2_ nanoparticles [38] and copper ions/nanoparticles act as antimicrobials when used to treat bacteria [39]. Finally, Dde_2342 is glyceraldehyde-3-phosphate dehydrogenase, which understandably would play a critical role in glycolysis as the cells metabolize endogenous reserves for ATP production.

### Proteins only accumulated in response to exposure to Pt^4+^ ions

Some proteins accumulated in the presence of Pt ions but not Pd ions and vice versa. Included in the platinum-specific dataset are Dde_2137, which is the small subunit of the NiFe hydrogenase, and Dde_2138, a *c*_3_-type cytochrome that is believed to donate electrons to the NiFe hydrogenase active site. The NiFe hydrogenase small subunit is transported into the periplasm by the twin-arginine targeting (TAT) pathway. Both of these proteins were shown to be required for metal ion reduction by *D. fructosovorans* [18]. The NiFe hydrogenase genes are flanked by two genes encoding the large and small subunits of an alternative NiFeSe hydrogenase and maturation proteins, but none of these other proteins were more abundant following incubation with Pt^4+^. Another, ferredoxin, Dde_2642, also accumulated during incubation with Pt^4+^ ions. This protein is thought to be involved in metal binding and electron transfer in the cytoplasm. Whether it is able to shuttle electrons to the platinum ions in *Desulfovibrio* is unknown.

The ability of platinum ions to cause DNA lesions that inhibit cell division is the basis for their chemotherapeutic use in drugs such as cisplatin. The single strand-binding (SSB) protein (Dde_1545) accumulated in bacteria incubated with Pt^4+^ ions [40]. Binding of Pt is prevented by the binding of SSB to single-stranded DNA. Two other potential stress response proteins accumulated during incubation with Pt^4+^ ions. One was rubrerythrin (Dde_1214), which, because of its ability to protect *D. vulgaris* against hydrogen peroxide damage, is suggested to protect against cytoplasmic oxidative stress [41, 42]; it was also previously found to be downregulated when using nitrate as an electron acceptor [43]. The other is a methyl-accepting chemotaxis sensory transducer (Dde_1279), which contains a PAS domain at the conserved-C-terminus that is likely to be part of a signal response to promote motility in the presence of platinum.

### Proteins only accumulated in response to exposure to Pd^2+^ ions

No obvious candidates for redox proteins required for Pd^2+^ reduction accumulated specifically in response to incubation with Pd^2+^ ions. Although carbon monoxide reductase (Dde_3028), the hybrid cluster protein (Hcp) (Dde_2640) and a quinone-linked NAD(P)H dehydrogenase (Dde_1618) were more abundant than in the control, these increases were just over 1.5-fold. The Hcp in particular has been implicated in response to nitric oxide stress in both *D. vulgaris* [44] and *E. coli* [45]. The four other proteins that increased significantly included a peptidase that might be involved in a stress response, but again the increases relative to the control were only about twofold. The Dde_2200 protein is a 1-deoxy-d-xylulose-5-phosphate synthase involved in the metabolism of pyruvate, a key intermediate in various metabolic pathways. Since the cells are in buffer (with palladium), it is likely that this would increase metabolism of what little pyruvate is available. Similarly, Dde_1699 and Dde_0444, both of which are zinc-containing peptidases, are also present and are likely to be degrading proteins that have misfolded due to the presence of the palladium/oxidative stress involved in metal reduction [46]. Clearly, however, most of the proteins induced in response to exposure to Pd^2+^ were those also induced in the presence of Pt^4+^ ions.

### Increase in nanoparticle sizes due to increased synthesis of the NiFe hydrogenase

A major aim of the proteomic experiments was to investigate whether proteins could be identified that when overexpressed might facilitate the controlled production of metal nanoparticles of a specific size. Initially, six proteins were targeted, (Dde_0806, Dde_1442, Dde_2315, Dde_0444, Dde_0168 and Dde_2137), of which two were unable to be sub-cloned into the pMO9075 plasmid into *E. coli* (Dde_0444, Dde_0806), and three gave no change in the resistance to their respective metals or alteration to the nanoparticles formed (data not shown). However, pMO9075, containing the gene encoding Dde_2137, was viable in both *E. coli* and *D. alaskensis* G20.

**Fig. 3.**
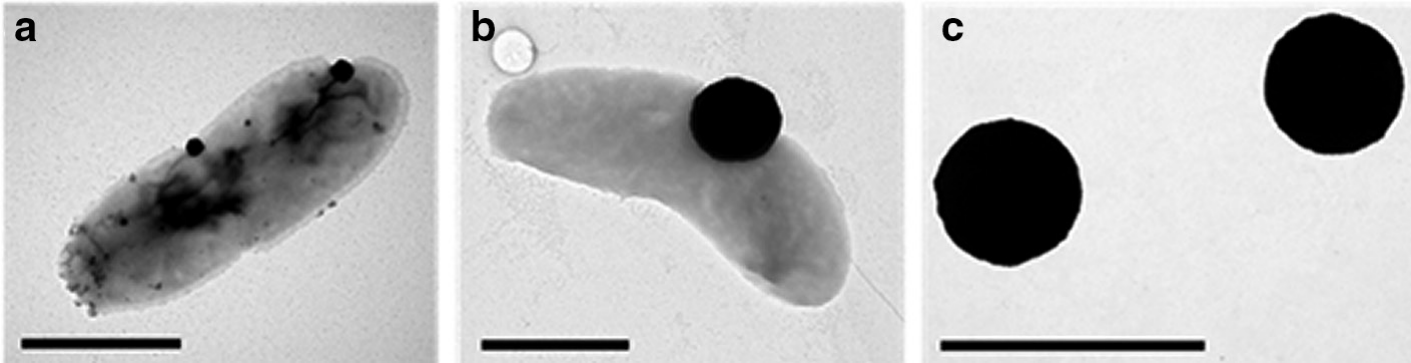
Electron micrographs of *D. alaskensis* incubated with 2 mM PtCl_4_ for 120 min. (a). Control containing the empty pMO9075 plasmid. (b). Containing the pMO-2137 plasmid. (c). The nanoparticles from (b) free in the media. Scale bar, 1 µm. Further images are available in Supplementary File 1.

**Fig. 4.**
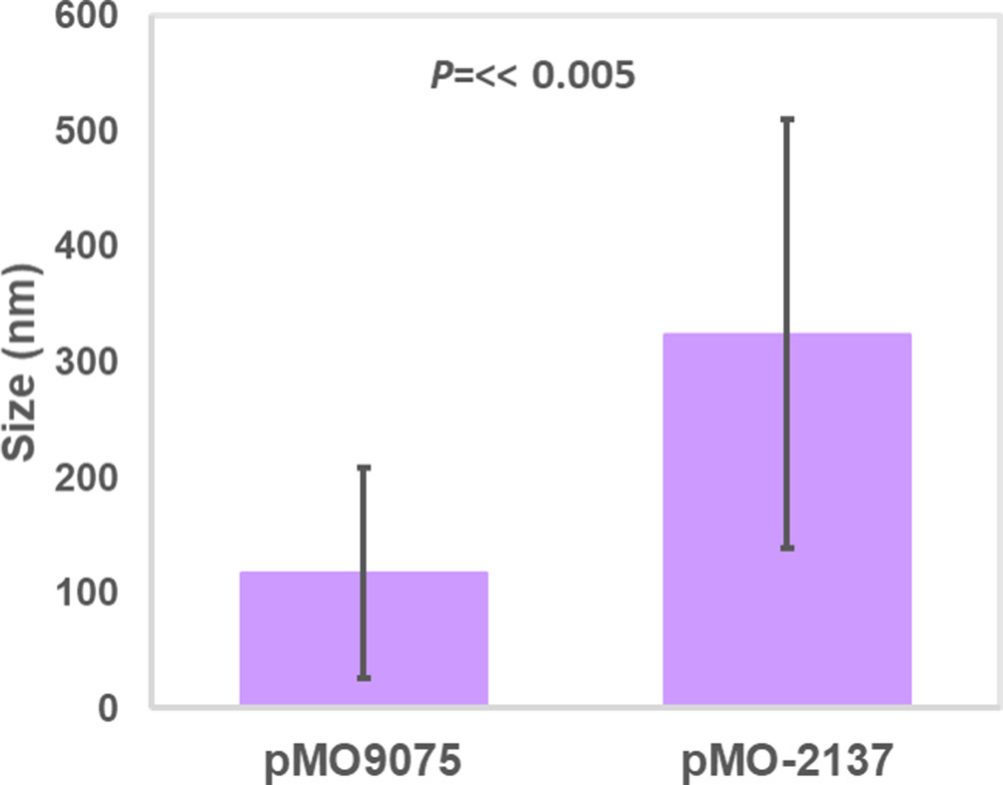
Size distribution of the platinum nanoparticles produced by *D. alaskensis* containing the two different plasmids, the pM09075 control and pM09075 containing the gene encoding Dde_2137. The error bars represent the standard deviation of the mean.

**Fig. 5.**
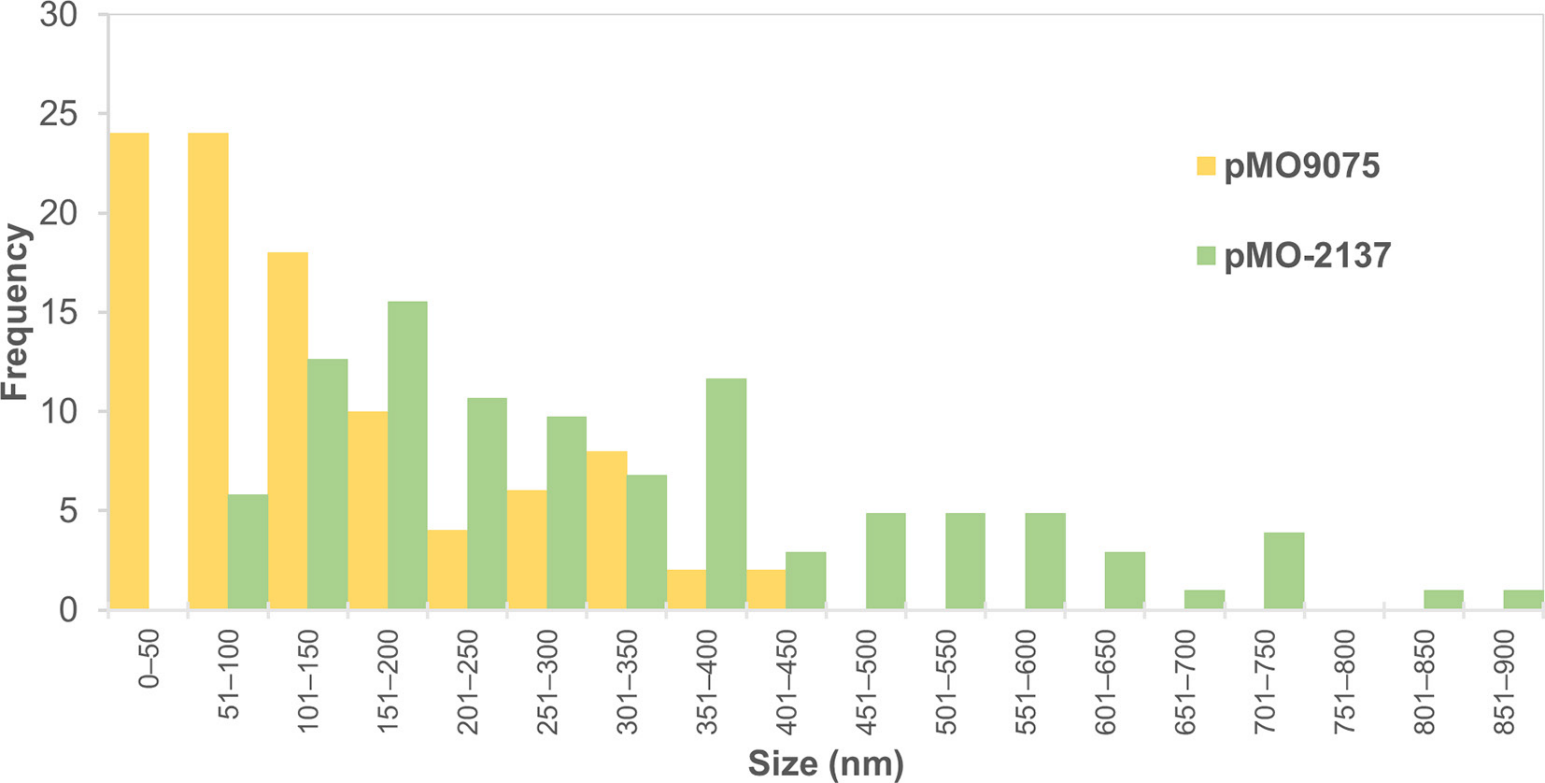
Frequency distribution of the sizes of the platinum nanoparticles produced by *D. alaskensis* containing either the control plasmid (pMO9075) or the one containing the gene dde_2137 encoding the small subunit of the NiFe hydrogenase (pMO-2137).

Given the previous demonstration that a mutant defective in NiFe hydrogenase synthesis was also defective in nanoparticle production [18], the observation that this enzyme accumulated during Pt^4+^ reduction was especially significant. We therefore designed experiments to determine whether overexpression of the NiFe hydrogenase would result in changes to the physical properties of the nanoparticles formed. The gene encoding the NiFe hydrogenase protein dde_2137 was cloned into the *D. alaskensis* plasmid pMO9075 to give pMO-2137. Introduction of this plasmid to *D. alaskensis* G20 significantly increased the size of the Pt nanoparticles when cells were exposed to platinum ions (*P*<<0.005) (Fig. 3). On average, the size increased from 117 nm for the empty plasmid control to 324 nm for the strain carrying the pMO-2137. Increased production of the NiFe protein also increased the range of nanoparticle sizes formed (Figs 4 and 5). Many of the larger particles were free in the buffer, not attached to the cell. Although quantification of the number of nanoparticles formed in the control versus the pMO-2137 strain was not carried out, it is expected that the number of particles formed will decrease in pMO-2137, as more platinum has been used to make each larger particle. Furthermore, there was no observed difference in the rate of reduction of platinum between the samples. Although larger nanoparticles were formed, the transformants were not more resistant to platinum treatment. We therefore suggest that the increased amount of Dde_2137 leads to increased deposition of platinum nanoparticles on the cell surface and, in turn, an increased localized turnover rate of the platinum ions by the autocatalytic abilities of the platinum nanoparticles, thus leading overall to larger nanoparticles. This supports previous work which concluded that the NiFe hydrogenase is required for nanoparticle synthesis in *Desulfovibrio* [19] and also their deposition on the outer membranes in *Shewanella* [21]. As the NiFe hydrogenase is predicted to be located in the periplasmic space, the ions, once reduced, must in some way be shuttled to the outside for deposition on the cell surface potential via either/both Dde_3627 and Dde_1415, the outer-membrane transporters found in both proteomic datasets. Deposition on the cell surface, possibly at the opening of the transporter, will allow the accumulation of more/large nanoparticles from further efflux or an autocatalytic effect from ions in the medium. Currently there are no potential proteins, either highlighted in this study or described in other published works, to account for how the nanoparticles are held on the cell surface, although, as the pMO-2137 are so readily ablated from the cell surface, it is likely that they are not held by very strong forces. Potentially, the LPS layer of the Gram-negative cell may have a role in the binding of the nanoparticle due to the outer layer’s weakly negative charge or simply through being entangling due to nature of the outer-side layer of the membrane [47].

**Fig. 6.**
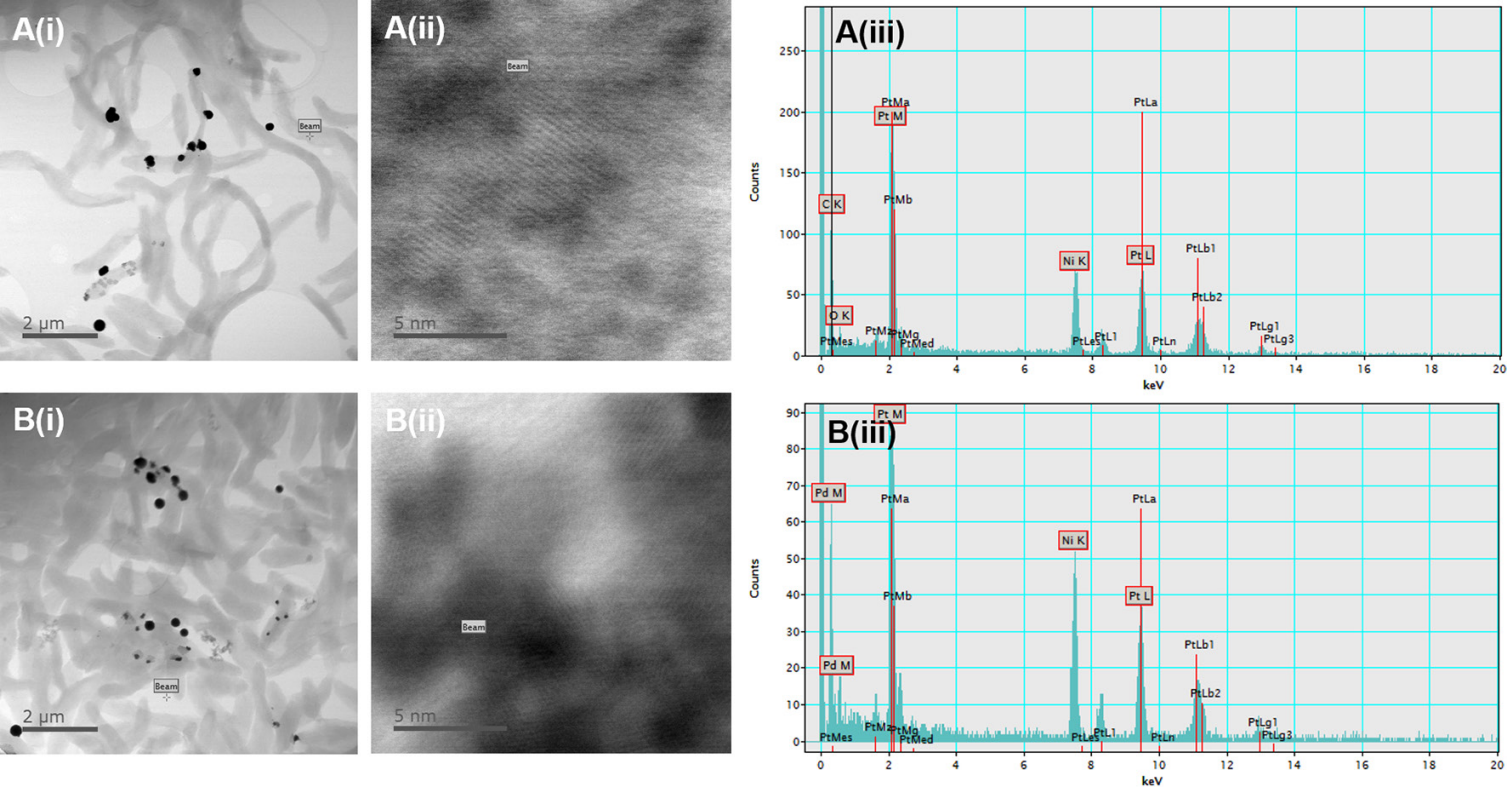
TEM and EDS spectra of *D. alaskensis* cells in the presence of platinum. (a) Cells containing the empty plasmid pMO9075. (b) Cells containing the pMO-2137 plasmid. (i) Low-magnification TEM image of the sample. (ii) TEM image of the area of analysis. (iii) Resultant EDS spectra of (ii). The beam indicates the place at which the EDS analysis was collected in (iii).

Samples of nanoparticles produced by *Desulfovibrio* transformed with either pMO-2137 or the empty vector control were analysed using energy-dispersive spectroscopy (EDS). In both cases the nanoparticles were shown to be entirely made of platinum, with some regions containing very regular atomic lattices (Fig. 6).

## Conclusions

Despite the fact that *D. alaskensis* has been used for metal ion bioremediation for some years, this paper, to the best of our knowledge, presents the first shotgun proteomic analysis of bacteria synthesizing palladium and platinum nanoparticles. Although various strains of *Desulfovibrio* were used for these previous studies, only a few of the proteins reported here have previously been implicated in metal reduction and nanoparticle production.

We have identified some proteins that accumulate during both palladium or platinum reduction and others that respond to one of the metals, but not the other. This implies that there are separate pathways for the reduction of each metal, but that there is also an underlying core pathway that features in the reduction of both. One possible reason for this is that Pt^4+^ ions are reduced via Pt^2+^ and that the divalent Pd^2+^ and Pt^2+^ cations are reduced by a common pathway (Fig. 2). Shared pathways for metal homeostasis are common in many bacteria, particularly for metals that have similar periodicity. For example, parts of the copper homeostasis pathway in *E. coli* are homologous to those of the silver pathway. The two metals not only induce the expression of parts of the pathway, but they also share efflux machinery [48, 49]. Similarly, p-type ATPases are common metal-ion shuttles from the cytoplasm to the periplasm in a wide range of bacterial resistance and toxicity mechanisms [50].

As there appears to be a central pathway for palladium and platinum homeostasis in *D. alaskensis *, this may have the potential to aid in the recovery of these metals from an industrial setting. One of the most common waste streams containing these metals is from spent catalytic convertors, which often contain a mixture of the two. Recovering these metals using a bacterium that has resistance pathways to both metals would significantly enhance this process, while any genetic modification to the pathway could potentially either target one particular metal or enhance the recapture of both.

The identification of both groups of these proteins presents the opportunity not only of increasing the removal of metals from platinum- and palladium-containing solutions, but also for increasing the specificity of the process. Genetic manipulation to control the expression of the genes for these target proteins might enable increased metal specificity, efficiency and even metal resistance to be achieved. In proof-of-principle experiments to test these ideas, we have also shown the feasibility of genetically engineering *D. alaskensis* to alter nanoparticle sizes. One aim of future work will be to develop the ability to produce nanoparticles tailored for specific needs as well as the application of what we have engineered to environmental samples.

Although increasing the size of the nanoparticles formed was not the target of the study, it does show there is precedent for changes at the genetic level to lead to altered characteristics for the nanoparticles formed. Increasing the size is not necessarily a useful trait in the formation of nanoparticles, as it lowers surface area-to-mass ratio, resulting in lower catalytic ability, but the practicalities of isolating larger particles does speed up the recovery process. It is possible that mutations to the Dde_2137 protein could produce smaller, more highly catalytic Pt nanoparticles, which would be more useful for applications rather than recovery. A recent study has also shown that *D. alaskensis* has the ability to produce bi-metallic nanoparticles made of both palladium and platinum and illustrates how they are useful for redox reactions applicable to industrial settings [16]. This further underpins there being a shared pathway for the formation of both the palladium and platinum nanoparticles, which potentially are effluxed using the same machinery (Dde_3627 and Dde_1415), as highlighted in this work. Using the pMO-2137 strain in the formation of nanoparticles in a bi-metallic setting could potentially also alter the reactivity of the nanoparticles by altering the ratio of the two metals in the nanoparticle.

One of the limiting factors for progressing this work further is the fact that there are few genetic tools available for use in *Desulfovibrio* sp. and genetic engineering is limited to large cumbersome plasmids without clear replication origins. There are no reports describing the use of CRISPR/Cas in the *Desulfovibrio* sp. and expression studies are limited, with even basic genetic reporters (such as GFP) not functioning in this bacterium. A more robust genetic toolkit will be required to further enhance the remit of *Desulfovibrio* sp. for its use in industrial applications and the recapture of metals lost to waste streams.

## Supplementary Data

Supplementary material 1

Supplementary material 2
