# Supplementary material for: Shotgun proteomic analysis of nanoparticle-synthesizing Desulfovibrio alaskensis in response to platinum and palladium
Source: Microbiology (Reading). 2019 Jul 30;165(12):1282–94. doi: 10.1099/mic.0.000840 (PMC7376266; doi:10.1099/mic.0.000840)
Supplement: Supplementary material 1 [file mic-165-1282-s001.pdf]

Supplementary File 1

Shotgun proteomics analysis of nanoparticle-synthesising *Desulfovibrio alaskensis* in response to platinum and palladium

Michael J. Capeness[a], Lisa Imrie [b], Lukas F. Mühlbauer[a], Thierry Le Bihan[b,c], Louise E. Horsfall[a*]

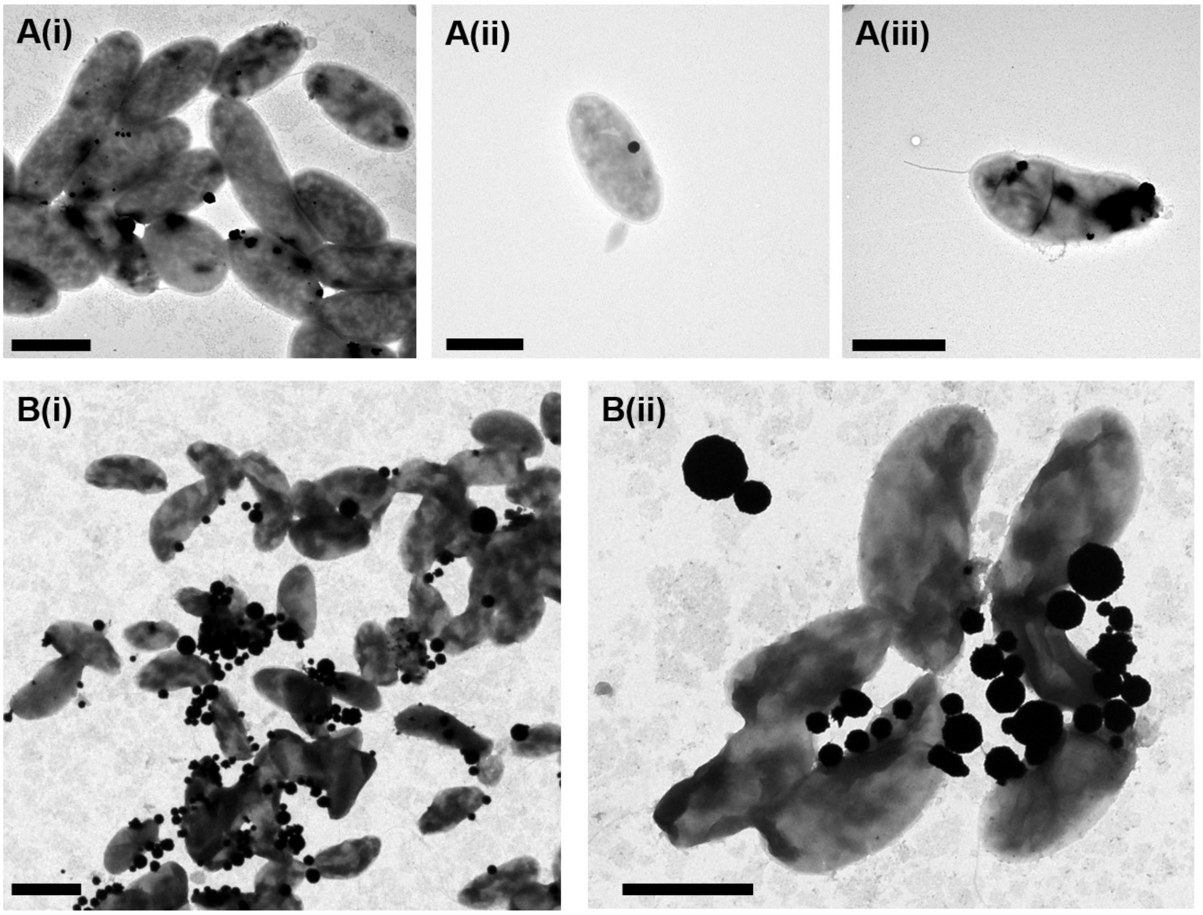

Supplementary Image 1. TEM images of the platinum nanoparticles synthesised by *D. alaskensis* harbouring the control plasmid pMO9075 (A) or pMO-2137 (B). Scale bars in all images = 1 µm.
